# Molecular Consequences of Depression Treatment: A Potential In Vitro Mechanism for Antidepressants-Induced Reprotoxic Side Effects

**DOI:** 10.3390/ijms222111855

**Published:** 2021-11-01

**Authors:** Przemysław Sołek, Jennifer Mytych, Anna Tabęcka-Łonczyńska, Marek Koziorowski

**Affiliations:** Department of Biotechnology, Institute of Biology and Biotechnology, University of Rzeszow, Werynia 2, 36-100 Kolbuszowa, Poland; annaurz@wp.pl (A.T.-Ł.); mkozioro@ur.edu.pl (M.K.)

**Keywords:** antidepressants, apoptosis, DNA methylation, male fertility, toxicity mechanisms

## Abstract

The incidence of depression among humans is growing worldwide, and so is the use of antidepressants. However, our fundamental understanding regarding the mechanisms by which these drugs function and their off-target effects against human sexuality remains poorly defined. The present study aimed to determine their differential toxicity on mouse spermatogenic cells and provide mechanistic data of cell-specific response to antidepressant and neuroleptic drug treatment. To directly test reprotoxicity, the spermatogenic cells (GC-1 spg and GC-2 spd cells) were incubated for 48 and 96 h with amitriptyline (hydrochloride) (AMI), escitalopram (ESC), fluoxetine (hydrochloride) (FLU), imipramine (hydrochloride) (IMI), mirtazapine (MIR), olanzapine (OLZ), reboxetine (mesylate) (REB), and venlafaxine (hydrochloride) (VEN), and several cellular and biochemical features were assessed. Obtained results reveal that all investigated substances showed considerable reprotoxic potency leading to micronuclei formation, which, in turn, resulted in upregulation of telomeric binding factor (TRF1/TRF2) protein expression. The TRF-based response was strictly dependent on p53/p21 signaling and was followed by irreversible G2/M cell cycle arrest and finally initiation of apoptotic cell death. In conclusion, our findings suggest that antidepressants promote a telomere-focused DNA damage response in germ cell lines, which broadens the established view of antidepressants’ and neuroleptic drugs’ toxicity and points to the need for further research in this topic with the use of in vivo models and human samples.

## 1. Introduction

According to the World Health Organization (WHO), depressive disorders are the greatest burden of disease in developed countries, affect millions of people, and are the leading cause of disability around the world [1]. Depression itself is characterized by a comprehensive impact on all aspects of human life, and may have a recurrent and chronic nature and a synergistic exacerbation effect in the presence of other conditions [2]. Antidepressants are the first-line agents for the initial treatment of depressive disorders. They were first developed in the 1950s. Currently, there are several different generations and classes of antidepressants. Their mechanism of action is generally associated with the chemical imbalance correction of neurotransmitters in the human brain [3,4]. However, the recent data point to alternative mechanisms, such as interaction with tropomyosin receptor kinase B [5].

Psychiatrists routinely use antidepressants to treat depression, making their application significant and growing worldwide [6]. In addition, usually, treatment is long-term and should be continued beyond the acute phase for patients to obtain the full therapeutic benefits [7,8]. Antidepressants have proven efficacy, but it is clear that good tolerance is the most important factor [9]. In turn, the dark side of pharmacotherapy is related to the side effects of antidepressants, also linked to human sexuality. Sexual dysfunctions have a significant impact on quality of life and recovery [10]. However, until now, clinicians were more concerned about other troublesome side effects. It turns out that sexual dysfunctions caused by antidepressants have important clinical consequences and are often the cause of non-compliance with therapeutic recommendations or premature discontinuation of treatment, which may lead to the recurrence of depressive symptoms [11,12,13]. In addition to the reduction of sexual interest, drugs can make it difficult to become aroused, sustain arousal, and reach orgasm, which ultimately results in a patient’s low self-esteem [14]. The frequency of male sexual dysfunction appears to be significantly lower with drugs which mechanisms of action involve the adrenergic or dopaminergic systems. However, the relationship between depressive disorders, pharmacotherapy, and sexual dysfunction is very complex and there is no mechanistic data to provide clinical guidelines [13,15].

The exact mechanism of the effect of antidepressants on human sexual function is unknown, but most of them modulate the concentration of central neurotransmitters such as serotonin, norepinephrine, and dopamine. Serotonin inhibits the production of nitric oxide, which physiologically plays a key role in the sexual response, and its elevated levels impair sexual function. Furthermore, norepinephrine and dopamine are also involved in the cycle of sexual response related to desire and arousal. Many antidepressants can also suppress normal sexual function through cholinergic and alpha-1 adrenergic receptors [14,16]. Importantly, sexual side effects may subside with a lower, but still therapeutic dose, therefore detailed studies on direct interaction of the antidepressants with the human reproductive cells are highly sought.

The purpose of this study was to determine the effect of selected antidepressants and neuroleptic drugs on mouse spermatogenic cells (GC-1 spg and GC-2 spd cell lines) and provide mechanistic data of cell-specific responses to antidepressant treatment. This preliminary study becomes an important aspect in terms of effectiveness for treating depression and will provide clinically relevant data associated with side effects of drugs and male fertility reduction.

## 2. Results

### 2.1. Antidepressants Interfere with ATP Production

**Figure 1:**
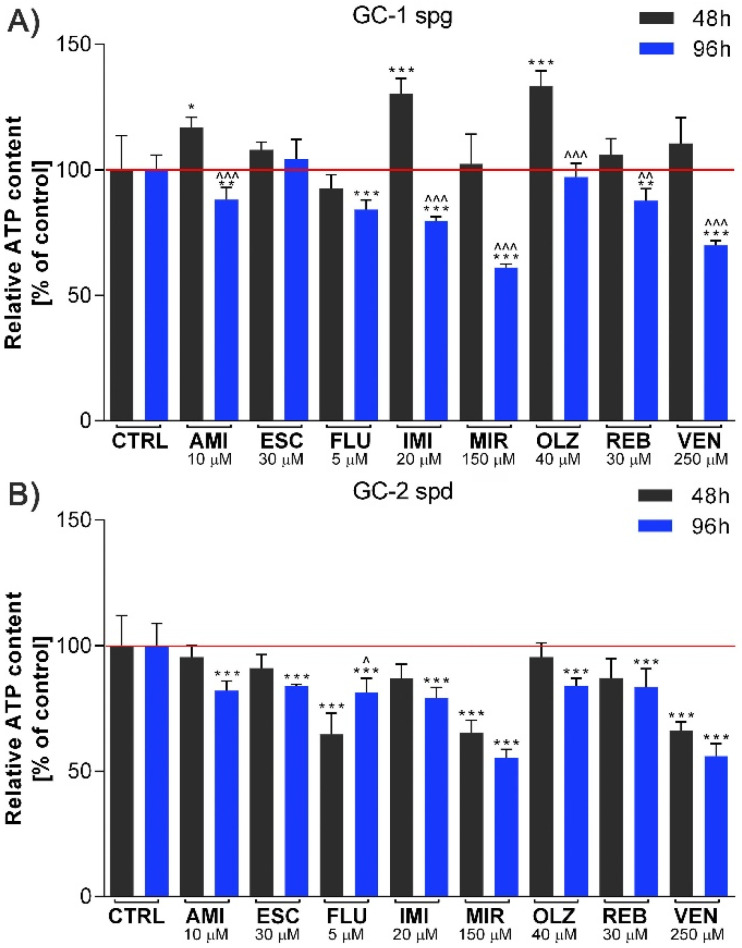
Antidepressants interfere with spermatogenic cells energy production. GC-1 spg (**A**) and GC-2 spd (**B**) cells were exposed to antidepressants for 48 or 96 h. The ATP production was then measured. Statistical differences were determined using one-way analysis of variance (ANOVA) with Dunnett’s post-hoc test; *p* values < 0.05 were considered statistically significant. Asterisks (*) indicate the comparison between control and antidepressants-treated cells, whereas carets (^) indicate the comparison between the same drugs in different periods (48 vs. 96). Bars indicate mean ± SD, *n* = 3, ***/^^^ *p* < 0.001, **/^^ *p* < 0.01, */^ *p* < 0.05, no indication–no statistical significance.

We first investigated the effect of different antidepressants on ATP production in GC-1 spg and GC-2 spd cells. Drug concentrations were selected based on our previous research. Chosen concentrations inhibited the MTT activity of tested cells by 50% [17]. After 48 h drug exposure, in only three experimental sets of the GC-1 spg cell line, we noted a statistically significant increase in ATP production (AMI, IMI, OLZ) (*p* < 0.05, *p* < 0.001). Interestingly, at the same time, the GC-2 spd cell line was characterized by a significant decrease in ATP level (FLU, MIR, VEN) (*p* < 0.001). The most pronounced decrease was noted for cells treated with MIR and VEN (both cell lines tested) after 96 h incubation (*p* < 0.001). For the rest of the drugs analyzed, a statistically significant decrease in the ATP was also observed after 96 h incubation. The exceptions were two drugs—ESC and OLZ (GC-1 spg)—where the ATP level remained unaffected (Figure 1A,B).

### 2.2. Antidepressant-Induced Cell Cycle Arrest and Related Protein Network Activation

**Figure 2:**
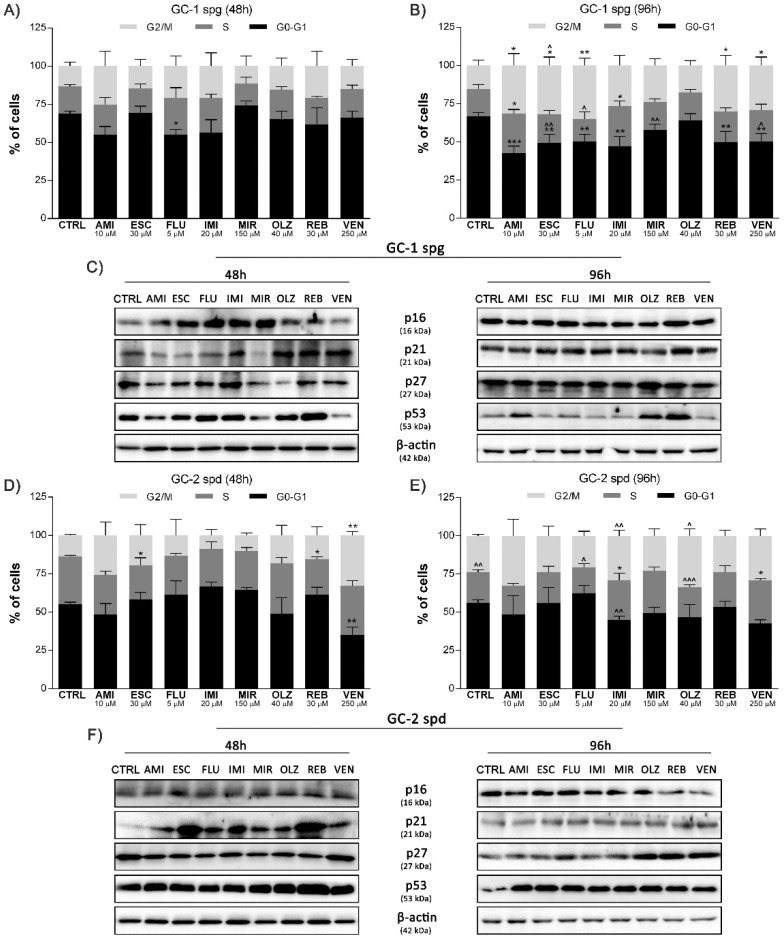
Antidepressant-induced cell cycle profile regulation through related genes activation. GC-1 spg and GC-2 spd cells were treated with antidepressants for 48 and 96 h, the cell cycle profile (**A**,**B**,**D**,**E**) was determined and the level of p16, p21, p27, p53 proteins was controlled by Western blot technique. Representative blots are shown (**C**,**F**). Statistical differences were determined using one-way analysis of variance (ANOVA) with Dunnett’s post-hoc test; *p* values < 0.05 were considered statistically significant. Asterisks (*) indicate the comparison between control and antidepressants-treated cells, whereas carets (^) indicate the comparison between the same drugs in different periods (48 vs. 96). Bars indicate mean ± SD, *n* = 3, ***/^^^ *p* < 0.001, **/^^ *p* < 0.01, */^ *p* < 0.05, no indication–no statistical significance.

In response to ATP depletion, cell cycle arrest was initiated in GC-1 spg and GC-2 spd cells after antidepressant treatment. Short-term incubation (48 h) caused a reduction in cell population in G0-G1 phase for FLU (*p* < 0.05) (GC-1 spg) and VEN (*p* < 0.01) (GC-2 spd). In turn, for ESC and REB, the S phase of the cycle was reduced (*p* < 0.05) (GC-2 spd only). Along with longer exposure to antidepressants, a significant reduction of the G0-G1 phase (*p* < 0.01–0.001) with a simultaneous extension of the S (*p* < 0.05) and G2/M phases (*p* < 0.05–0.01) (GC-1 spg) was observed for most experimental sets. A similar trend was not confirmed for the GC-2 spd cell line (Figure 2A,B,D,E). Moreover, cell cycle progression is controlled by various molecules. In the case of the GC-1 spg cell line, we noted a total increase in the level of all analyzed proteins, i.e., p16, p21, p27, and p53, regardless of the incubation time (*p* < 0.05–0.001). The second line, in general, also showed an increase in the synthesis level of these proteins after 48 and 96 h (*p* < 0.05–0.001) of incubation with drugs (Figure 2C,F and Supplementary File).

### 2.3. Antidepressants Initiate the DNA Fragmentation of Spermatogenic Cells

**Figure 3:**
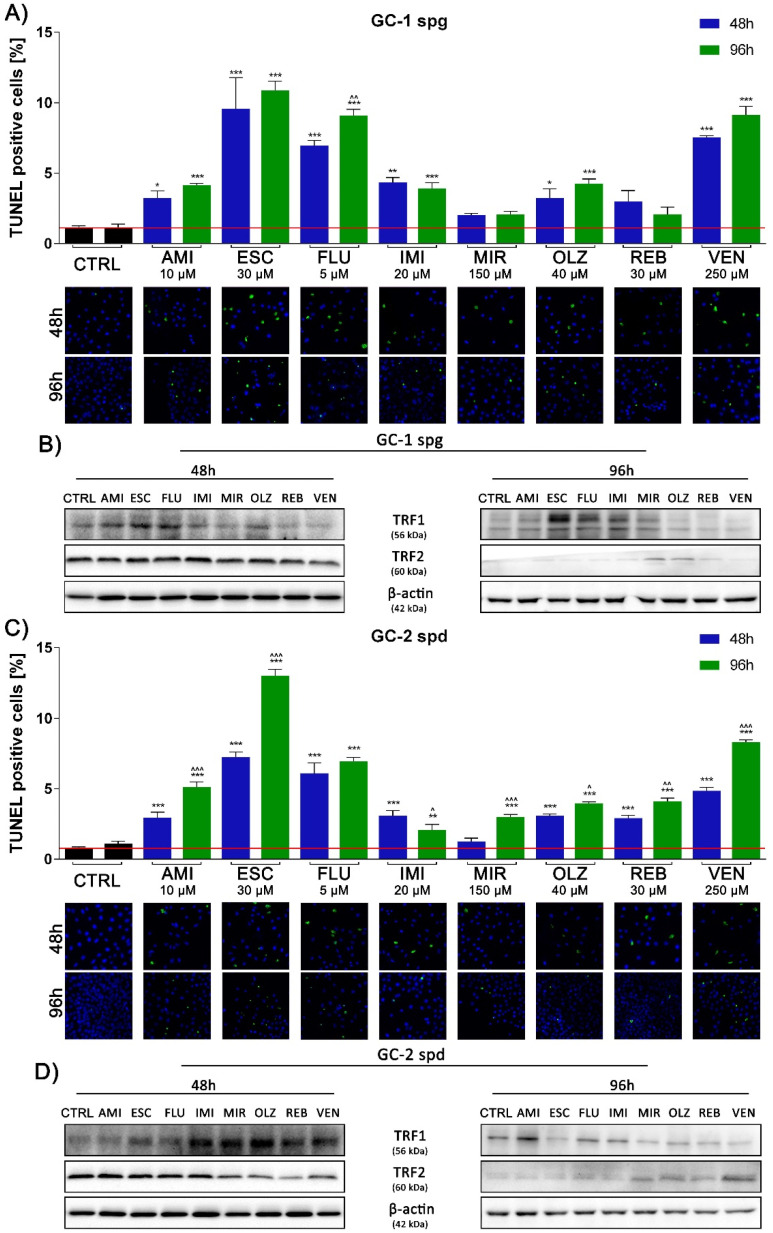
Toxic concentrations of antidepressants promote DNA fragmentation of treated cells. GC-1 spg and GC-2 spd cells were treated with antidepressants for 48 and 96 h. The TUNEL labeling system to detect the DNA damage was then applied (**A**,**C**) and the level of TRF1, TRF2 proteins were controlled by Western blot technique. Representative blots (**B**,**D**) and photos of TUNEL cells are shown (magnification 10×). Blue fluorescence (DAPI) live cells, green fluorescence (FITC) apoptotic cells. Statistical differences were determined using one-way analysis of variance (ANOVA) with Dunnett’s post-hoc test; *p* values < 0.05 were considered statistically significant. Asterisks (*) indicate the comparison between control and antidepressants-treated cells, whereas carets (^) indicate the comparison between the same drugs in different periods (48 vs. 96). Bars indicate mean ± SD, *n* = 3, ***/^^^ *p* < 0.001, **/^^ *p* < 0.01, */^ *p* < 0.05, no indication–no statistical significance.

We then assessed the fragmentation of the 3′-OH DNA ends that are generated during apoptotic cell death. Our results show an increase in TUNEL positive cells in all experimental sets, regardless of the time of exposure, and studied cell lines. The number of apoptotic cells increased with the incubation time (both lines tested). The highest number was noted for ESC, FLU, and VEN in all experimental sets. Only MIR and in part REB drugs were characterized by the percentage of TUNEL + cells at the control level (Figure 3A,C). In addition, the previously noted irreversible cell cycle arrest could be caused not only by DNA fragmentation but also by damage to the telomeric regions at the ends of the chromosomes. Indeed, we observed a total increase in the TRF1 and TRF2 protein expression in both cell lines tested after 48 and 96 h of incubation. The only exception was the GC-1 spg cell line and the incubation time of 48 h, where a significant decrease in TRF1 level was observed for the OLZ, REB, and VEN. Similarly, for TRF2 and 96 h incubation time for AMI, ESC, FLU, IMI, and VEN (Figure 3B,D and Supplementary File).

### 2.4. Antidepressants Induce Micronuclei Formation in Spermatogenic Cells with No Effect on Methylation Status

**Figure 4:**
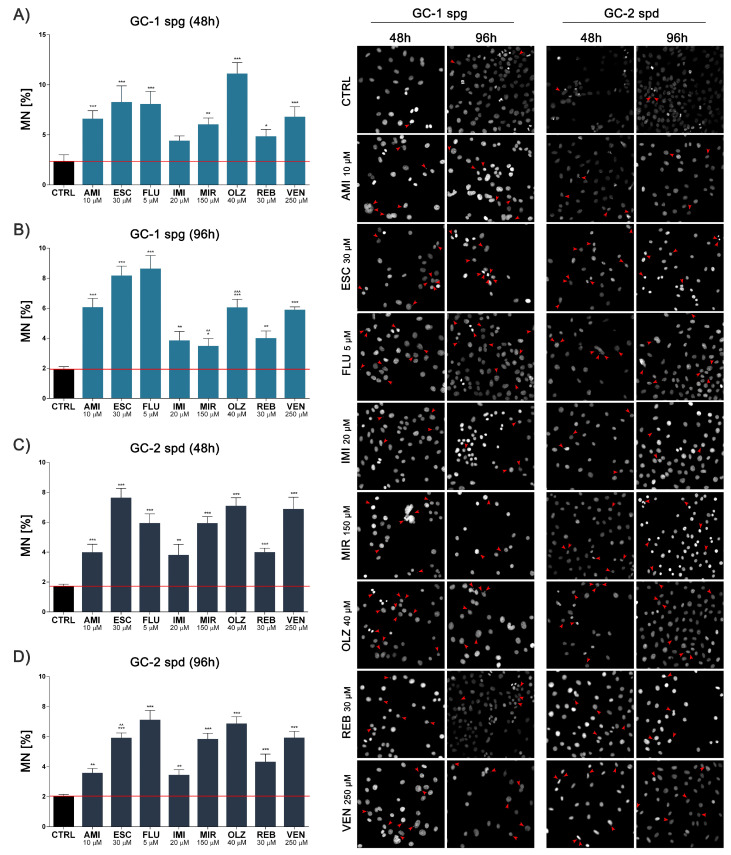
Antidepressants increase the frequency of micronuclei formation. Mouse spermatogenic cells were treated for 48 and 96 h and then the effects of antidepressants on micronuclei formation (MN) were evaluated (**A**–**D**). Representative photos are shown; red arrows indicate MN (magnification 10×). Statistical differences were determined using one-way analysis of variance (ANOVA) with Dunnett’s post-hoc test; *p* values < 0.05 were considered statistically significant. Asterisks (*) indicate the comparison between control and antidepressants-treated cells, whereas carets (^) indicate the comparison between the same drugs in different periods (48 vs. 96). Bars indicate mean ± SD, *n* = 3, ***/^^^ *p* < 0.001, **/^^ *p* < 0.01, * *p* < 0.05, no indication–no statistical significance.

**Figure 5:**
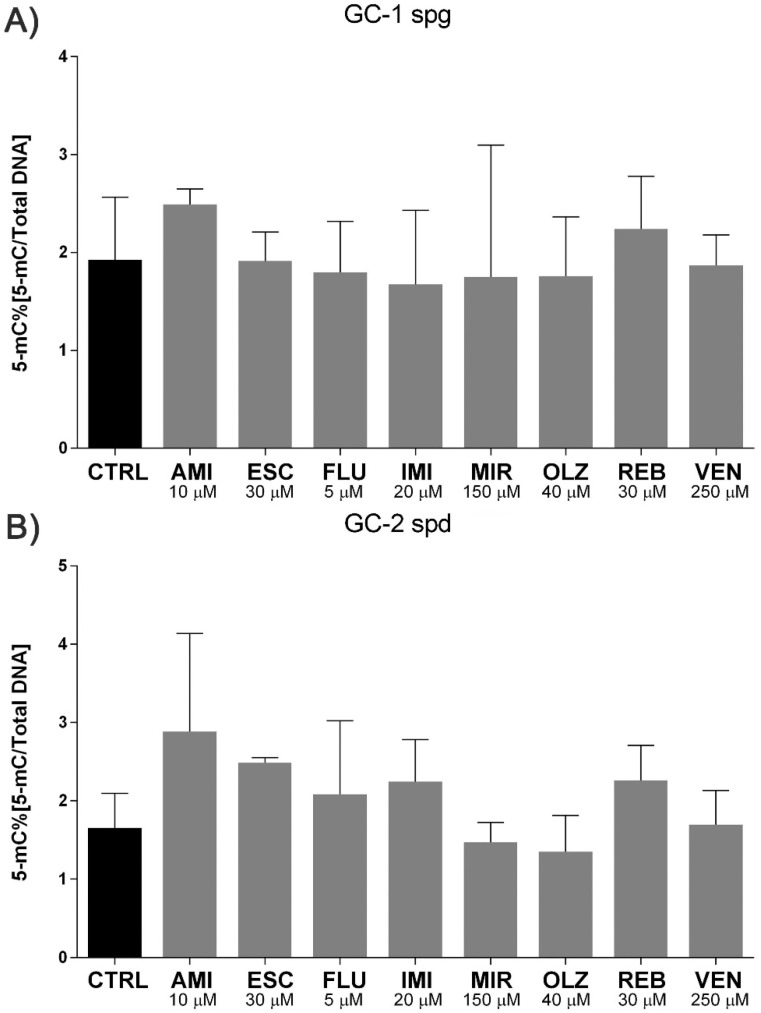
Antidepressants do not alter the methylation DNA amount. Mouse GC-1 spg and GC-2 spd cells were treated for 48 and 96 h and then the effects of antidepressants on global DNA methylation status were evaluated (**A**,**B**). Statistical differences were determined using one-way analysis of variance (ANOVA) with Dunnett’s post-hoc test; *p* values < 0.05 were considered statistically significant, no indication–no statistical significance.

Persistent stress can lead to further damage to the nucleic acids. We noted a significant increase in micronuclei production after 48 h (except IMI) and 96 h incubation for both cell lines tested. Interestingly, after 96 h exposure, we observed a slight decrease in the amount of MN compared to 48 h, and this trend was observed for all experimental sets (Figure 4A–D). Simultaneously, no differences in DNA methylation pattern, as assessed by 5-mC levels, were observed (Figure 5A,B).

### 2.5. Antidepressants Affect NuMa-Tubulin Interactions

**Figure 6:**
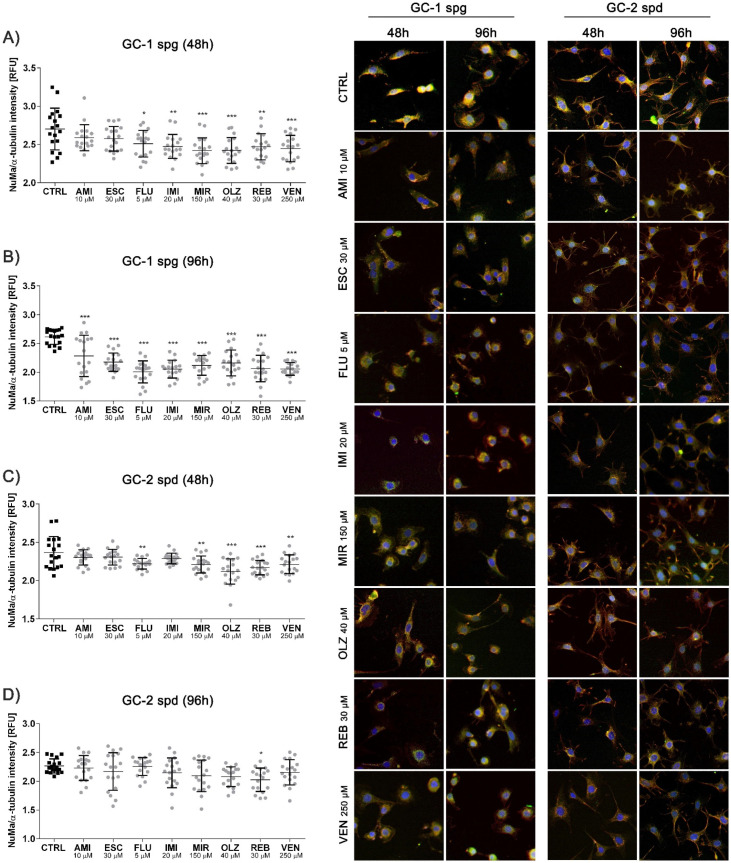
Antidepressants interact between the nuclear mitotic apparatus protein and α-tubulin. Mouse spermatogenic cells were treated for 48 and 96 h and then the immunofluorescence analysis of the interaction between protein stabilizing structures of microtubule-centromeres (NuMa, α-tubulin) at the spindle abnormal formation process were evaluated (**A**–**D**). Representative photos are shown (magnification 10×). Green fluorescence (FITC) NuMa, red fluorescence (Texas Red) α-tubulin, blue fluorescence (DAPI) nucleus. Statistical differences were determined using one-way analysis of variance (ANOVA) with Dunnett’s post-hoc test; *p* values < 0.05 were considered statistically significant. Asterisks (*) indicate the comparison between control and antidepressants-treated cells. Bars indicate mean ± SD, *n* = 18, *** *p* < 0.001, ** *p* < 0.01, * *p* < 0.05, no indication–no statistical significance.

Next, we assessed the interaction between nuclear mitotic apparatus protein (NuMa) and α-tubulin. We noted a decrease in NuMa/α-tubulin ratio intensity in most experimental sets of the GC-1 spg cells (48 and 96 h) except AMI and ESC (48 h) (Figure 6A,B). A similar trend was observed for GC-2 spd cells after 48 h incubation with antidepressants, however, with less significance and except AMI, ESC, IMI (48 h) (Figure 6C). Interestingly, the GC-2 spd cells were unaffected after longer incubation in most cases (Figure 6D).

### 2.6. Antidepressants Reduce Spermatogenic Cells Viability

**Figure 7:**
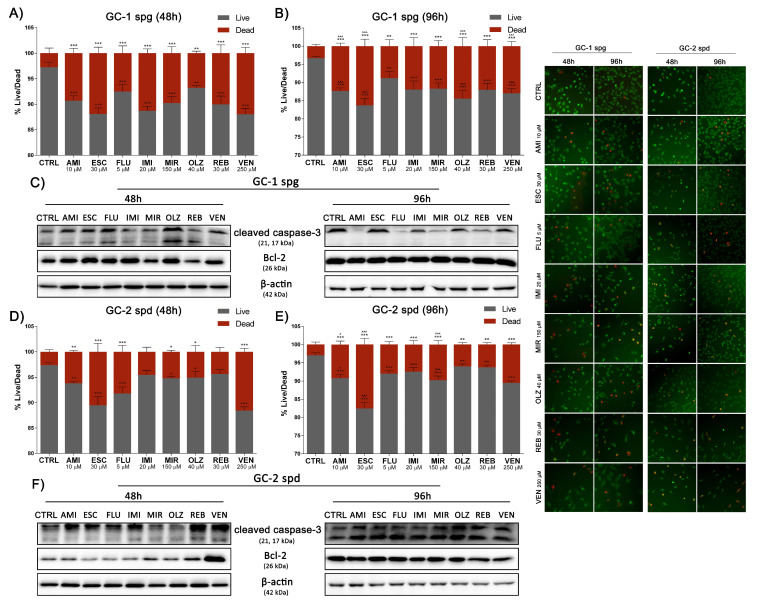
Antidepressants reduce cell viability and induce apoptosis. Mouse GC-1 spg and GC-2 spd cells were treated with antidepressants for 48 and 96 h, subjected to simultaneous fluorescence staining of viable and dead cells (**A**,**B**,**D**,**E**) and then the level of cleaved caspase-3, Bcl-2 was controlled by Western blot technique. Representative blots (**C**,**F**) and photos of Calcein/PI staining are shown (magnification 10×). Green fluorescence (FITC) live cells, red fluorescence (Texas Red) dead cells. Statistical differences were determined using one-way analysis of variance (ANOVA) with Dunnett’s post-hoc test; *p* values < 0.05 were considered statistically significant. Asterisks (*) indicate the comparison between control and antidepressants-treated cells, whereas carets (^) indicate the comparison between the same drugs in different periods (48 vs. 96). Bars indicate mean ± SD, *n* = 3, ***/^^^ *p* < 0.001, **/^^ *p* < 0.01, */^ *p* < 0.05, no indication–no statistical significance.

Finally, we have conclusively confirmed cell death resulting from exposure to antidepressants. We observed a significant decrease in cell viability after 48 h incubation with drugs for the GC-1 spg (all sets). The effect was even more pronounced after 96 h of exposure (Figure 7A,B). In turn, the GC-2 spd cell line was characterized by higher resistance and thus a lower percentage of dead cells after 48 h. Interestingly, also for this cell line after 96 h incubation, significant decreases in cell viability were observed in all experimental sets (Figure 7D,E). Analysis of the differential staining results showed an increase in the number of dead cells along with the duration of the culture time. The highest percentage of dead cells after 96 h incubation compared to short-term culture was observed for AMI, ESC, OLZ, VEN (GC-1 spg), and AMI, ESC, MIR (GC-2 spd) sets. At the same time, we noted an increase in the cleavage of caspase 3 after 48 h in the GC-1 spg cell line (ESC, FLU, OLZ, VEN sets). This state was also maintained after 96 h exposure, except for FLU, where the level of cleaved caspase 3 decreased significantly, and analogous results were observed for the AMI, MIR, and REB. For the second cell line, GC-2 spd, an increase in the pool of cleaved caspase 3 was noted in all sets. At the same time, the level of Bcl-2 expression remained unaffected (GC-1 spg) or decreased significantly (GC-2 spd) except for VEN (48 h) and AMI (96 h) (Figure 7C,F and Supplementary File).

## 3. Discussion

Most authors agree that sexual dysfunction is a possible side effect of pharmacological treatment. Toxicity to reproductive cells is fundamental due to the possibility of genetic changes in the cells of future generations. Therefore, the toxicity of antidepressants becomes an important issue in the context of treatment efficacy. Evidence suggests that the toxic concentrations of antidepressants can affect redox imbalances and disrupt the cellular energy status. We here confirm such assumptions related to the total decrease in ATP production after 48 and 96 h of treatment. The obtained results may indicate a progressive impairment of the mitochondrial function and thus confirm our previous observations. The side effect of antidepressants and neuroleptic drugs may be related to the influence on ATP synthesis by oxidative phosphorylation inhibition in mitochondria and interaction with phospholipids in the inner mitochondrial membrane or with sulfhydryl groups present in the sperm cell membrane. Mitochondrial ATP is essential for Ca^2+^ reuptake, which leads to their overload and cell death [18,19,20].

Under stress, cell cycle arrest often occurs. As a result, cells can repair all necessary damage. The assessment of the cell cycle profile changes showed a reduction of the G0-G1 phase, with a simultaneous extension of the S and G2/M phases for the GC-1 spg cell line. In contrast, the second line of GC-2 spd showed no major changes. Thus, it is the first evidence of the positive effects of repair and activation of adaptation mechanisms to stress conditions. Others also indicate a cycle arrest in the G2/M phase or S, suggesting that antidepressants can exhibit cytotoxic properties and induce apoptotic cell death possibly via a mitochondrial independent pathway [21,22]. In turn, cell cycle arrest in the G0-G1 phase may be associated with the inhibition of cyclin D2 activity in a dose-dependent manner. Such observations are very important as D-type cyclin proteins are critical factors that regulate the proper course of the cell cycle from the G1 to S phase [23]. Here, we also noticed the activation of proteins (p16, p21, p27, p53). All of the analyzed proteins are involved in the molecular regulation of the cell cycle profile by inhibition of cyclin-dependent kinase (CDK) (p16), inhibition of DNA replication initiation by CDK complex (p21), regulation of CDK activity at the G1/S phase transition (p27), and provision of controlled cell division (p53) [24]. Activation of these proteins may again indicate an attempt to effectively repair DNA damage leading to the proliferation or activation of adaptive mechanisms. These assumptions may also be supported by the fact that spermatogenesis is one of the most complex and long-term processes of cell proliferation and differentiation [25,26,27]. Misregulation of the cell cycle may lead to impaired development of germ cells, embryonic cell apoptosis, and decreased fertility [28]. In addition, p21 and p27 proteins play a key role in the self-renewal and differentiation of testicular stem cells [29]. Others also confirm that antidepressants regulate the differentiation and proliferation of various cell types through mechanisms of glucocorticoid receptor (GR) phosphorylation and GR-dependent upregulation of p27 and p57 proteins [30]. Further studies confirm direct cell cycle arrest as the effect of antidepressants on non-spermatogenic cells [31,32]. The possible reasons for such results could be inhibition of ERK1/2 kinase phosphorylation, decreased expression of c-fos, c-jun, cyclin A, cyclin D1, and increased expression of the p21/p53 pathway genes [33].

High levels of oxidative stress can damage the DNA of reproductive cells in patients treated with antidepressants [34] and here, we observed a significant increase in cellular DNA fragmentation and micronuclei formation in all experimental sets. Undoubtedly, this may be related to the results so far and indicates a decrease in mitochondrial potential in ATP production and possibly irreversible damage to these cellular structures. Antidepressant treatment was linked with disturbances in mitotic and meiosis processes, as well as chromosomal aberrations [35,36,37] resulting in total sperm count reduction, motility disorders, and anomalies in the morphological shapes [38,39]. Also, NuMa plays a key role in the organization and stabilization of the mitotic spindle apparatus. The protein is highly dependent on the phase of the cell cycle, and its distribution is regulated by phosphorylation and dephosphorylation [40]. Others reported abnormal mitotic spindle formation after exposure to the various drugs. Moreover, some cytotoxic drugs interact with tubulin subunits leading to disturbance of polymerization and depolymerization of microtubules and directly impairing the function of the mitotic spindle [41].

In addition to DNA breaks, damage to the telomeric sections at the ends of chromosomes may also be associated with cell cycle arrest. Consequences of telomere dysfunction included genomic instability, apoptosis, or cellular senescence [42]. Research indicates a relationship between the levels of TRF1 and TRF2 proteins, expression of p53 and MAPK kinase, and the induction of apoptosis [43]. Based on this information, we noted a total increase in the synthesis of TRF1 and TRF2 in both cell lines treated with antidepressants. Interestingly, only in GC-2 spd cells, the level of TRF1 and TRF2 remained significantly up-regulated compared to control after 96 h of exposure. Such a result indicates the type-dependent cell response to antidepressant treatment. Few studies provide evidence of telomere length reduction in patients with depressive disorders [44,45] and changes in the amount of mitochondrial DNA (mtDNA) or telomere length as a consequence of stress and depression [46]. However, there are no data regarding changes in TRF1 and TRF2 expression after antidepressant treatment, thus the obtained results allow us to propose a completely new mechanism of the spermatocytes apoptosis pathway associated with these two proteins.

In the last step, we noted activation of cell death mechanisms in all test sets related to upregulation of caspase 3 cleavage and decreased amounts of Bcl-2 synthesis. The interaction of proteins from the Bcl-2 family is crucial for cell death or survival. Moreover, the Bcl-2 family mediates the process by which mitochondria contribute to cell death, called the intrinsic pathway of apoptosis [47]. Other studies confirm our results [48,49]. Patients with depressive disorders are characterized by low expression of the anti-apoptotic Bcl-2, but treatment with antidepressants increased Bcl-2 levels. Also, preclinical studies have shown that antidepressant treatment induces elevated Bcl-2 levels and protects against apoptotic cell death by interaction with the mitochondrial voltage-dependent anion channels [50,51].

Apoptosis is a necessary process that plays an important role in various physiological processes and occurs with high frequency in the male reproductive system, particularly in the testis. It seems that apoptosis occurs continuously in the testicular tissue because mammals’ spermatogenesis is a complex process that requires precise homeostasis of various cell types [52]. There are no mechanistic data on the induction of apoptosis in spermatocyte cells by antidepressants. However, studies indicate that antidepressants cause DNA fragmentation, lipid peroxidation resulting in free radicals’ release, which disrupts the cell membrane, and then significantly damages the tissues [48,49,53].

At this point, it is crucial to mention the several limitations of this study. The study was done on spermatogenic cells and presents the mechanistic data limited to the in vitro system. The testicular tissues are known for their extreme complexity and thus the obtained data need to be further confirmed in in vivo animal systems. However, the presented results are important molecular data pointing directly to the exact mechanism associated with reprotoxic potential of antidepressants commonly used in clinical practice. Also, the toxicity mechanism has been proposed based on the general data obtained for several antidepressants and neuroleptic drugs tested.

**Figure 8:**
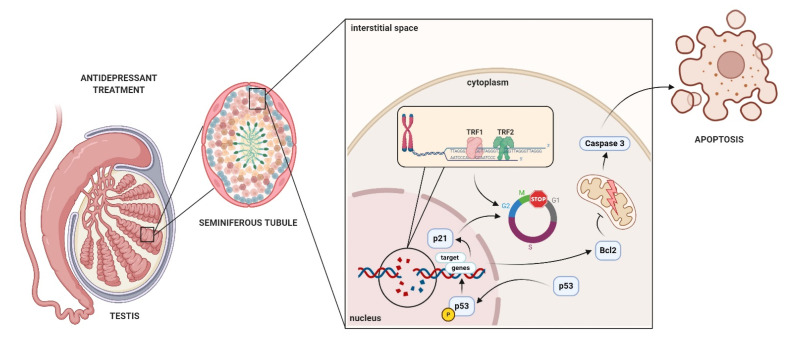
A potential mechanism for antidepressants-induced reprotoxic side effects. The antidepressant and neuroleptic drugs treatment induce the genotoxic events and adaptation mechanisms in GC-1 spg and GC-2 spd cells.

In conclusion, antidepressants and neuroleptic drugs are characterized by a delayed onset of therapeutic action, which requires long-term use. The use of this type of drug may be associated with side effects (Figure 8). Few reports also indicate that this may result in delayed various disorders of life functions, including reproductive functions and processes. Here, for the first time to our knowledge, we provide molecular evidence of possibly irreversible disorders in the course of spermatogenesis by germ cell damage.

## 4. Materials and Methods

### 4.1. Chemicals

The chemicals were analytical or biotechnology grade and were used without further modification. Unless stated otherwise, reagents and drugs were obtained commercially from Sigma-Aldrich (Poznan, Poland), Thermo Fisher Scientific (Waltham, MA, USA), or Cayman Chemicals (Ann Arbor, MI, USA).

### 4.2. Cell Culture

Mouse-derived spermatogonia (GC-1 spg, ATCC, CRL-2053, Manassas, VA, USA) and spermatocyte (GC-2 spd, ATCC, CRL-2196, Manassas, VA, USA) cell lines were cultured in complete growth medium DMEM for mammalian cells, supplemented with 10% fetal bovine serum (FBS, EURx, Poland) and 1% antibiotic mix solution of 100 U/mL penicillin, 0.1 mg/mL streptomycin, and 29.2 mg/mL L-glutamine (Thermo Fisher Scientific, Waltham, MA, USA). Cells were maintained at standard conditions 37 °C and 5% CO_2_ in a humidified environment and passaged by trypsinization every three days (confluency ~80%). For all experiments, cells were seeded at an optimal density of 3.0 × 10^3^ cells/cm^2^.

### 4.3. Drug Application

Drugs were dissolved in high purity solvent DMSO (dimethyl sulfoxide) to a 100 mM stock solution according to characteristic solubility. Further dilutions were prepared before the experiment by serial dilutions of the stock solution in a complete DMEM medium. Next, dilutions were added to the culture vessels in the chosen concentration depending on the experiment.

### 4.4. ATP Luminescence Assay

The ATP luminescence assay for quantitative evaluation of cell proliferation was performed according to the manufacturer’s protocol (PerkinElmer, Waltham, MA, USA). Cells were seeded in a 96-well plate at a density of 1.0 × 10^3^ cells/well. After 24 h, cells were treated with drugs at selected concentrations and incubated for 48 and 96 h. The culture medium was then removed and lysis solution was added. Plates were shaken for 10 min and a substrate solution was pipetted. After 15 min of incubation in the dark, the luminescence was measured in a multi-detection reader (PerkinElmer Victor X4 2030, USA). The results are given as % of cellular ATP, and the control value is taken as 100%.

### 4.5. Cell Cycle Profile and Micronuclei Formation

Cells were seeded on black 96-well fluorescent plates at a density of 1.0 × 10^3^ cells/well for 24 h. Then, cells were treated with selected drug concentrations for 48 and 96 h, washed with DPBS solution, and fixed in 3.7% formaldehyde (20 min, RT) followed by adding 100 µL of Hoechst 33258 fluorescent dye (1 µg/mL) (10 min, RT). Pictures were taken with the use of InCell Analyzer 2000. The cell cycle assessment was performed by fluorescence intensity measurement using ImageJ software with a DNA Cell Cycle plug-in. Results were presented as % of G0/G1, S, and G2/M phases of total cell count. Based on the same images, the quantification of micronuclei formation was performed and the results were given as the number of micronuclei expressed in %.

### 4.6. TUNEL Apoptotic Cell Detection

Detection of DNA breaks was performed with the use of the DeadEnd Fluorometric TUNEL System (Promega, Madison, WI, USA) according to the manufacturer’s protocol. Briefly, cells were seeded at standard density for 24 h. Drugs at selected concentrations were then added for 48 and 96 h. Afterward, cells were fixed in 4% paraformaldehyde (20 min, RT) and positive control was performed by digestion with DNase I (3 U/mL in 50 mM Tris-HCl, pH 7.5, 1 mg/mL BSA) (10 min, 37 °C). Next, cells were washed with DPBS and covered with a mixture of the recombinant enzyme and the nucleotide mix and placed in a humid chamber at 37 °C for 60 min. Further DAPI dye was applied. Visualization and analysis were performed using InCell Analyzer 2000 (blue–DAPI, green–FITC). TUNEL positive cells were presented as % of total cells.

### 4.7. Cell Death Assays by Calcein-AM/PI Staining

Cells were routinely seeded on 96-well fluorescent plates. After 24 h of incubation, cells were treated with drugs and left 48 and 96 h for exposure. After this time, the cell viability assessment procedure was performed using the Fluo Cell Double Staining Kit according to the manufacturer’s instructions (MoBiTec, Berkheim, Germany). Cells were probed with a dye mixture of 1 mM calcein-AM and 1.5 mM propidium iodide in PBS for 15 min at 37 °C. Signal detection was performed using an In Cell Analyzer 2000 on two channels: red fluorescence–Texas Red and green fluorescence–FITC. The results were reported as cell viability in %.

### 4.8. Western Blotting

Proteins of interest were visualized by Western blotting according to Mytych et al. 2017 [54]. Briefly, cells were seeded at standard density, treated with drugs, and suspended in an ice-cold RIPA buffer supplemented with protease inhibitor. Protein content was determined by the BCA quantitation assay using BSA as a calibration. Next, 30 µg of samples were fractionated by 10% SDS-PAGE and electrotransferred from a polyacrylamide gel to PVDF membranes. Unspecific protein-binding sites were then blocked by incubation in 1% BSA and probed with the specific primary antibodies: anti-β-actin (1:10,000; #PA1-16889, RRID: AB_568434), anti-p16 (1:1000, #PA1-16639; RRID: AB_568662), anti-p21 (1:1000, #701151; RRID: AB_2532411), anti-p27 (1:1000, #PA5-13254; RRID: AB_2078006), anti-p53 (1:1000, #700439; RRID: AB_2532324), anti-TRF1 (1:1000, #MA1-46089; RRID: AB_1018574), anti-TRF2 (1:1000, #MA1-41001; RRID: AB_2201326) (Thermo Fisher, Waltham, MA, USA), anti-Bcl-2 (1:500, #sc7382; RRID: AB_626736) (Santa Cruz, CA, USA), and anti-active caspase 3 (1:2000, #NBP1-45435, RRID: AB_10008902) (Novus Biologicals, Briarwood, USA). Membranes were then washed and incubated with secondary HRP-conjugated antibodies: anti-mouse (1:40,000; #A9044, RRID: AB_258431) and anti-rabbit (1:40,000; #A0545, RRID: AB_257896) (Sigma, Poznan, Poland). Proteins were visualized by the chemiluminescent substrate (BioRad, Hercules, CA, USA) using a Fusion Fx7 system (Viber Lourant, Collegien, France) according to the provided instructions.

### 4.9. Methylation Status

The global DNA methylation status was estimated using MethylFlash Methylated DNA 5-mC Quantification Kit (Epigentek, Farmingdale, NY, USA) strictly following the manufacturer’s protocol. Briefly, cells were seeded at standard density, treated with selected drugs, and suspended in DNA isolation buffer. The DNA purification was performed using the Gentra Puregene Cell Kit according to the manufacturer’s instructions (Qiagen, Hilden, Germany). Next, 100 ng of DNA was bound to assay wells and the reaction was continued by 5-mC detection with the use of a kit. The percentage of methylated DNA in total DNA was calculated using the following formula: 5-mC% = [(sample OD–negative control OD)/(slope * DNA amount /ng/)] × 100%.

### 4.10. Immunofluorescent Staining

The immunostaining was done accordingly to Mytych et al. 2017 [16]. Briefly, cells were seeded at standard density, treated with antidepressant drugs, fixed with 4% formaldehyde, permeabilized with 0.1 % Triton-X, blocked with 1% BSA, and incubated with primary antibody: anti-NuMa (1:500, #PA1-32451, RRID: AB_2154615) and anti-alpha tubulin (1:500, #A11126, RRID: AB_2534135) (Thermo Fisher, Waltham, MA, USA). The secondary antibodies used were anti-rabbit Texas Red (1:100, #T-2767, RRID: AB_2556776) and anti-mouse Cy3 (1:100, #A10521, RRID: AB_2534030) (Thermo Fisher, Waltham, MA, USA). Nuclei were visualized with Hoechst 33258. Images were captured with InCell Analyzer 2000 and analyzed with analyzing module.

### 4.11. Statistical Analysis

All statistical analyses were carried out using Microsoft Excel and GraphPad Prism 6. The results are presented as the mean ± standard deviation of at least three independent experiments. The data were normalized separately for 48 h and 96 h experimental set-ups, with the control group being considered as 100%. The calculations were performed with a one-way analysis of variance and Dunnett’s posttest. The significance level was set at *p*-value of <0.05 and considered as statistically significant (***/^^^ *p* < 0.001; **/^^ *p* < 0.01; */^ *p* < 0.05). (*) indicates a comparison between non-treated and antidepressants-treated cells, and (^) indicates a comparison between the same drugs in different periods (48 h, 96 h).

## 5. Conclusions

In conclusion, antidepressants are characterized by a delayed onset of therapeutic action, which requires long-term use. The use of this type of drug may be associated with side effects. Few reports also indicate that this may result in delayed various disorders of life functions, including reproductive functions and processes. Here, for the first time to our knowledge, we provide molecular evidence of possibly irreversible disorders in the course of spermatogenesis by germ cell damage.

## Data Availability

The data that support the findings of this study are available upon request from the corresponding author (P. Solek).

## Supplementary Materials

The following are available online at https://www.mdpi.com/article/10.3390/ijms222111855/s1.
