# Supplementary figures and images for: Molecular Consequences of Depression Treatment: A Potential In Vitro Mechanism for Antidepressants-Induced Reprotoxic Side Effects
Source: Int J Mol Sci. 2021 Nov 1;22(21):11855. doi: 10.3390/ijms222111855 (PMC8584852; doi:10.3390/ijms222111855)

## GC-1 spg

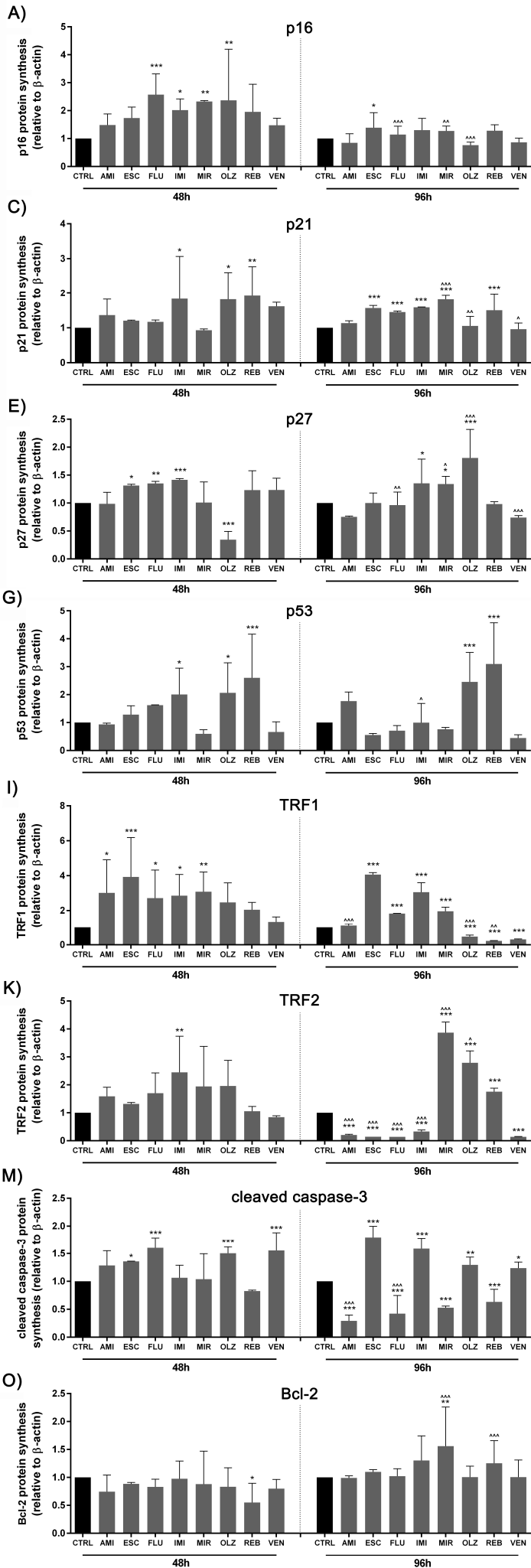

## GC-2 spd

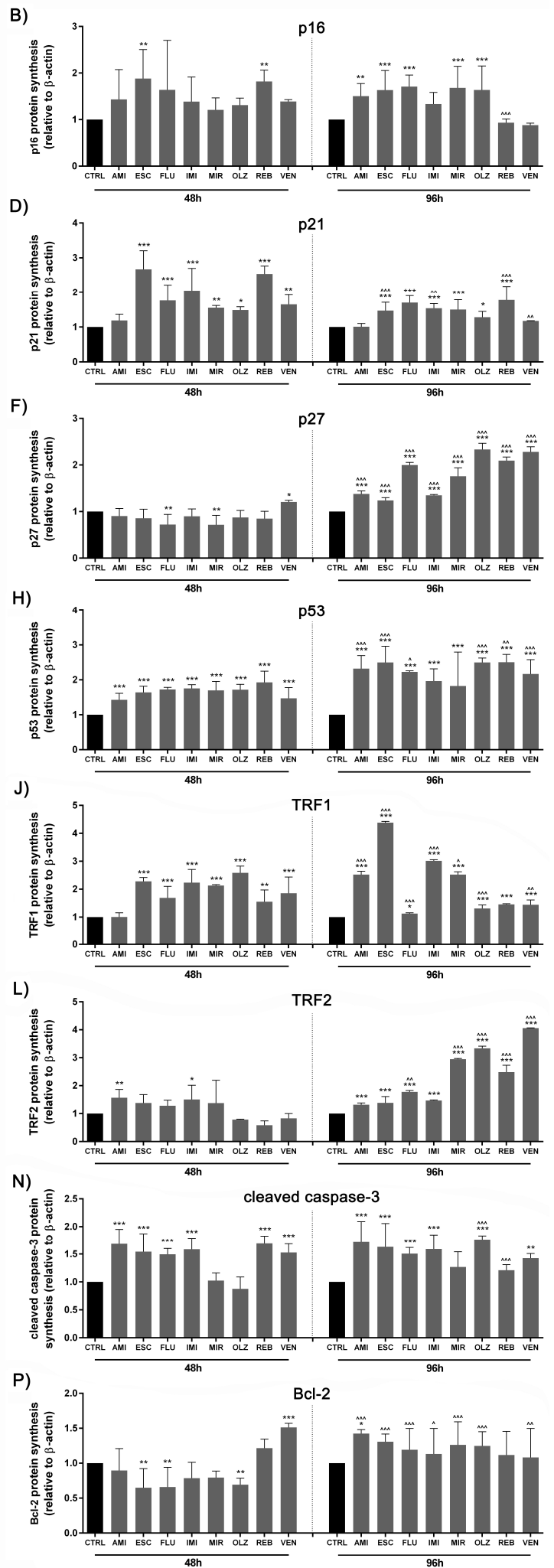

Supplement: Supplementary file 1 [file ijms-22-11855-s001.zip › ijms-1427811-SI.pdf]
